# D-dimer levels and outcomes in heart failure with mildly reduced ejection fraction

**DOI:** 10.1016/j.ijcha.2026.101915

**Published:** 2026-03-29

**Authors:** Finn Kronberg, Tobias Schupp, Michael Behnes, Michelle Goertz, Marielen Reinhardt, Noah Abel, Alexander Schmitt, Felix Lau, Mohammad Abumayyaleh, Thomas Bertsch, Ibrahim Akin, Kathrin Weidner

**Affiliations:** a Department of Cardiology, Angiology, Haemostaseology and Medical Intensive Care, University Medical Centre Mannheim, Medical Faculty Mannheim, Heidelberg University, Germany; b Institute of Clinical Chemistry, Laboratory Medicine and Transfusion Medicine, Nuremberg General Hospital, Paracelsus Medical University, Nuremberg, Germany

**Keywords:** Heart failure with mildly reduced ejection fraction, HFmrEF, D-dimer, Biomarkers

## Abstract

**Objective:**

The study investigates the influence of D-dimer levels on the prognosis of patients hospitalized with heart failure with mildly reduced ejection fraction (HFmrEF).

**Background:**

The prognostic impact of D-dimer levels has not yet been investigated in patients with HFmrEF.

**Methods:**

We retrospectively included consecutive patients with HFmrEF at one institution from 2016 to 2022. Patients were divided in quartiles according to their D-dimer levels, further sub-analyses were performed after excluding patients with conditions associated with increased D-dimer levels. The primary endpoint was all-cause mortality at 30 months (median follow-up), key secondary endpoint was the risk of heart failure (HF)-related rehospitalization.

**Results:**

In total, 1126 patients with HFmrEF were included with a median D-dimer level of 0.87 µg/mL (interquartile range 0.42–2.19 µg/mL). Higher D-dimer levels were associated with an increased risk of all-cause mortality (Q4 vs. Q1: hazard ratio (HR) 6.817; 95% confidence interval (95% CI) 4.401–10.561, p = 0.001), which was still observed after multivariable adjustment (Q4 vs. Q1: adjusted (a)HR 3.228; 95% CI 1.901–5.479; p = 0.001). This association was still observed after excluding patients with conditions associated with elevated D-dimer levels.

**Conclusion:**

Elevated D-dimer levels were independently associated with a higher risk of all-cause mortality in HFmrEF patients.

## 1 Introduction

Heart failure (HF) affects more than 64 million people worldwide, with rising incidence and high mortality despite ongoing improvements in cardiovascular care [1]. Among all HF patients, up to 25% suffer from HF with mildly reduced ejection fraction (HFmrEF, left ventricular ejection fraction (LVEF) 41–49%) [2]. The long-term mortality rate of HFmrEF remains high with 8% 1-year mortality and up to 75% 5-year mortality after HF-related hospitalization, in line with a risk of HF-related rehospitalization at 17% at 1 year [3], [4], [5].

Biomarkers are established tools for risk stratification in patients with HF [6]. Amino-terminal prohormone of brain natriuretic peptide (NT-proBNP) levels were demonstrated to correlate with higher left ventricular filling pressures, and were demonstrated to predict the risk of HF-related rehospitalization. This association was also demonstrated for high-sensitivity cardiac troponin (hs-cTn) levels [7], [8]. High-sensitive C-reactive protein (CRP) reflects systemic inflammation in patients with HF and is associated with worse outcome [9], [10]. Another widely-used and easily available biomarker associated with inflammation is D-dimers. D-dimers are soluble cleavage products formed during the breakdown of cross-linked fibrin by plasmin. They are a specific marker for the activation of coagulation and fibrinolysis and are routinely used as a laboratory parameter for the exclusion diagnosis of venous thromboembolism (VTE), such as deep vein thrombosis (DVT) and pulmonary embolism (PE) [11]. D-dimers are also linked to the systemic inflammatory response that occurs in HF [12], [13]. Whereas the prognostic impact of D-Dimer levels was yet investigated in patients with heart failure with preserved (HFpEF) and reduced LVEF (HFrEF) [14], data investigating the prognostic value of D-dimer levels in HFmrEF remain scarce.

Therefore, the present study sought to evaluate the prognostic value of D-dimer levels in patients hospitalized with HFmrEF.

## 2 Methods

### 2.1 Study patients, design and data collection

For the present study, all consecutive patients hospitalized with HFmrEF at one University Medical Centre were included from January 2016 to December 2022 [15]. Using the electronic hospital information system, all relevant clinical data related to the index event were documented, such as baseline characteristics, vital signs on admission, prior medical history, prior medical treatment, length of index hospital and intensive care unit (ICU) stay, laboratory values, data derived from all non-invasive or invasive cardiac diagnostics and device therapies, such as echocardiographic data, coronary angiography, data being derived from prior or newly implanted cardiac devices and HF-related pharmacotherapies at index hospital discharge. Every re-visit at the outpatient clinic or rehospitalizations related to HF or adverse cardiac events were documented until the end of the year 2022. The present study derived from the “Heart Failure With Mildly Reduced Ejection Fraction Registry” (HARMER), representing a retrospective single-center registry including consecutive patients with HFmrEF hospitalized at the University Medical Center Mannheim (UMM), Germany (clinicaltrials.gov identifier: NCT05603390). The registry was carried out according to the principles of the declaration of Helsinki and was approved by the medical ethics committee II of the Medical Faculty Mannheim, University of Heidelberg, Germany (ethical approval code: 2022–818).

### 2.2 Inclusion and exclusion criteria

**Fig. 1:**
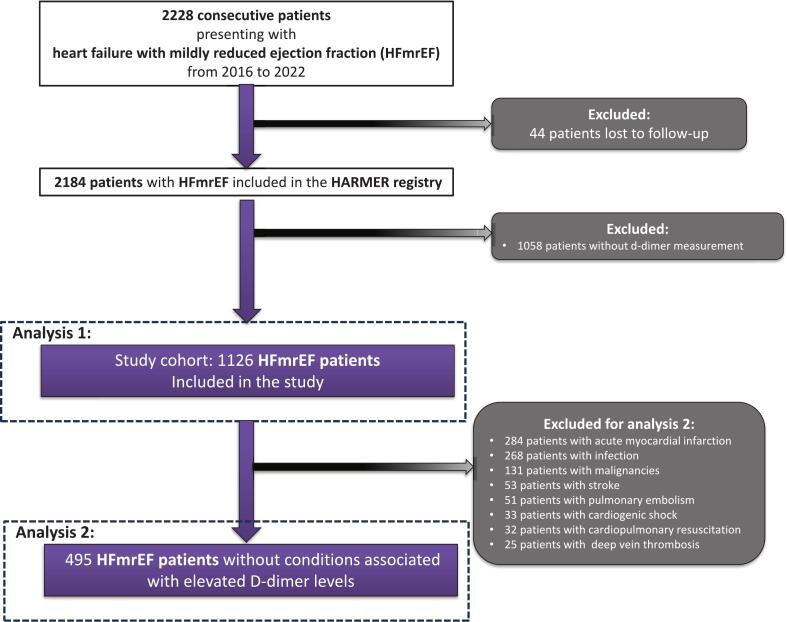
Study flow chart.

All consecutive patients with ≥ 18 years of age hospitalized with HFmrEF at one institution were included. The diagnosis of HFmrEF was determined according to the “2021 ESC Guidelines for the diagnosis and treatment of acute and chronic HF” [16]. Accordingly, all patients with LVEF 41–49% and symptoms and/or signs of HF were included. Transthoracic echocardiography was performed by cardiologists during routine clinical care being blinded to the final study analysis in accordance with current European guidelines [17]. Day 0 was defined as the day of index echocardiography. In patients with acute decompensated HF or concomitant myocardial infarction, echocardiography was performed following hemodynamic stabilization. The presence of elevated NT-proBNP levels and other evidence of structural heart disease were considered to make the diagnosis more likely, but were not mandatory for diagnosis of HFmrEF. For the present study, D-dimer testing was based on clinical decision-making during routine clinical practice and to the discretion of the treating physician. Patients without documented D-dimer levels were excluded from all analyses. No further exclusion criteria were applied for analysis 1. Within analysis 2, patients with comorbidities associated with elevated D-dimer levels such as malignancies, acute myocardial infarction (AMI), stroke, PE, DVT, infection, cardiopulmonary resuscitation (CPR) or cardiogenic shock were additionally excluded (Fig. 1**; Flowchart**) [18], [19].

### 2.3 Risk stratification, D-dimer measurement

The aim of the study was to investigate the prognostic impact of elevated D-dimer levels in HFmrEF. Therefore, we divided the cohort into quartiles (1st quartile (Q1), 2nd quartile (Q2), 3rd quartile (Q3), 4th quartile (Q4)) according to their D-dimer levels. In patients with multiple D-dimer testing we considered the one nearest to the time of diagnosis of HFmrEF by echocardiography. An immunoturbidimetric assay (Sysmex CS 5100, distributed by Siemens Healthineers, Erlangen, Germany) was used to quantify D-dimer concentrations in citrate plasma. Plasma was separated from sodium citrate whole blood (9NC, Sarstedt, Nürmbrecht, Germany) by centrifugation in accordance with the manufacturer's instructions. The extended linearity range of the assay spans between 0.19 and 80 µg/mL.

### 2.4 Study endpoints

The primary endpoint was all-cause mortality during median follow-up of 30 months. Secondary endpoints comprised in-hospital all-cause mortality, all-cause mortality at 12 months, rehospitalization for worsening HF, cardiac rehospitalization, AMI, stroke, coronary revascularization and major adverse cardiac and cerebrovascular events (MACCE) during follow-up. HF-related hospitalization was defined as a rehospitalization due to worsening HF requiring intravenous diuretic therapy. Cardiac rehospitalization was defined as rehospitalization due to a primary cardiac condition, including worsening HF, AMI, coronary revascularization and symptomatic atrial or ventricular arrhythmias. MACCE was defined as composite of all-cause mortality, coronary revascularization, non-fatal AMI and non-fatal stroke.

### 2.5 Statistical methods

Quantitative data were presented as mean ± standard error of mean (SEM), median and interquartile range (IQR), and ranges depending on the distribution of the data. They were compared using the Student’s *t* test for normally distributed data or the Mann-Whitney *U* test for nonparametric data. The Spearman-test was used for correlation. Deviations from a Gaussian distribution were tested by the Kolmogorov-Smirnov test. Qualitative data were presented as absolute and relative frequencies and were compared using the Chi-square test or the Fisher’s exact test, as appropriate. The prognostic impact of D-dimer levels stratified by quartiles was investigated using Kaplan-Meier analyses regarding the risks of all-cause mortality and HF-related rehospitalization at 30 months. For the secondary endpoint HF-related rehospitalization, patients experiencing the primary endpoint were censored at the time of death. Univariable hazard ratios (HR) were given together with 95% confidence intervals (CI). The prognostic impact of D-dimer levels was thereafter investigated within multivariable Cox regression models.

Results of all statistical tests were considered significant for p ≤ 0.05. SPSS (Version 28, IBM, Armonk, New York) was used for statistics.

## 3 Results

### 3.1 Study population

From 2016 to 2022, 2,228 patients with HFmrEF were hospitalized at our institution, of those 44 patients with incomplete follow-up were excluded. Another 1,058 patients without D-dimer measurement during index hospitalization were additionally excluded. Therefore, 1126 patients were included in analysis 1 with a median D-dimer level of 0.87 µg/mL (mean: 2.34 µg/mL; IQR 0.42–2.19 µg/mL).

Patients in the upper quartiles were older (median age 77 years in Q4 vs. 64 years in Q1, p = 0.001) and more likely to suffer from decompensated HF in the last 12 months (11.0% in Q4 vs. 8.5% in Q1, p = 0.041) chronic kidney disease (CKD) (36.3% in Q4 vs. 15.9% in Q1, p = 0.001), peripheral artery disease (PAD) (14.2% in Q4 vs. 2.2% in Q1, p = 0.001), stroke (19.6% in Q4 vs. 8.1% in Q1, p = 0.002), malignancy (19.2% in Q4 vs. 4.4% in Q1, p = 0.001), chronic obstructive pulmonary disease (COPD) (14.9% in Q4 vs. 8.1% in Q1, p = 0.016) and diabetes mellitus (40.2% in Q4 vs. 29.6% in Q1, p = 0.015). Patients with higher D-dimer levels suffered less often from hyperlipidaemia (33.6% in Q4 vs. 38.1% in Q1, p = 0.030) and were less often active smoker (34.5% in Q4 vs. 47% in Q1, p = 0.006). On admission, they presented more often with acute decompensated HF (36.7% in Q4 vs. 11.9% in Q1, p = 0.001) but less often with acute myocardial infarction (ST-elevation myocardial infarction (STEMI), 11.5% in Q1 vs. 7.8% in Q4, p = 0.010, Non-STEMI (NSTEMI), 17.4% in Q1 vs. 13.5% in Q4, p = 0.027) and therefore less coronary angiography was performed (65.2% in Q1 vs. 35.2% in Q4, p = 0.001) (Table 1**,** Table 2).

**Table 1:** Baseline characteristics.

	**Q1** (*n* = 270)		**Q2** (*n* = 294)		**Q3** (*n* = 281)		**Q4** (*n* = 281)		**p value**
**Age**, median (IQR)	64 (54–76)		75 (65–82)		79 (68–84)		77 (67–84)		**0.001**
**Male sex**, n (%)	197	(73.0)	195	(66.3)	173	(61.6)	172	(61.2)	**0.012**
**Body mass index,** kg/m^2^, median (IQR)	28.0 (24.8–32.1)		27.8 (24.9–31.5)		26.5 (23.4–30.9)		25.9 (22.9–29.8)		**0.001**
**SBP**, mmHg, median (IQR)	144 (130–161)		145 (129–168)		143 (124–161)		140 (120–160)		0.088
**DBP**, mmHg, median (IQR)	84 (74–95)		80 (70–96)		84 (68–98)		75 (65–88)		**0.001**
**Heart rate**, bpm, median (IQR)	80 (70–95)		80 (69–96)		84 (68–98)		84 (70–101)		0.422
**Medical history**, n (%)									
Coronary artery disease	107	(39.6)	151	(51.4)	128	(45.6)	103	(36.7)	**0.002**
Prior myocardial infarction	64	(23.7)	96	(32.7)	75	(26.7)	66	(23.5)	**0.045**
Prior PCI	82	(30.4)	119	(40.5)	90	(32.0)	69	(24.6)	**0.001**
Prior CABG	16	(5.9)	32	(10.9)	31	(11.0)	31	(11.0)	0.114
Prior valvular surgery	14	(5.2)	12	(4.1)	11	(3.9)	13	(4.6)	0.885
Congestive heart failure	91	(33.7)	125	(42.5)	111	(39.5)	96	(34.2)	0.085
Decompensated heart failure < 12 months	23	(8.5)	41	(13.9)	45	(16.0)	31	(11.0)	**0.041**
Prior ICD	6	(2.2)	8	(2.7)	3	(1.1)	5	(1.8)	0.532
Prior sICD	2	(0.7)	4	(1.4)	0	(0.0)	0	(0.0)	0.362
Prior CRT-D	6	(2.2)	7	(2.4)	5	(1.8)	3	(1.1)	0.659
Prior Pacemaker	19	(7.0)	26	(8.8)	34	(12.1)	20	(7.1)	0.119
Chronic kidney disease	43	(15.9)	105	(35.7)	129	(45.9)	102	(36.3)	**0.001**
Peripheral artery disease	6	(2.2)	30	(10.2)	36	(12.8)	40	(14.2)	**0.001**
Stroke	22	(8.1)	41	(13.9)	41	(14.6)	55	(19.6)	**0.002**
Liver cirrhosis	2	(0.7)	3	(1.0)	7	(2.5)	11	(3.9)	**0.029**
Malignancy	12	(4.4)	23	(7.8)	42	(14.9)	54	(19.2)	**0.001**
COPD	22	(8.1)	33	(11.2)	46	(16.4)	42	(14.9)	**0.016**
**Cardiovascular risk factors,** n (%)									
Arterial hypertension	195	(72.2)	235	(79.9)	227	(80.8)	215	(76.5)	0.066
Diabetes mellitus	80	(29.6)	123	(41.8)	102	(36.3)	113	(40.2)	**0.015**
Hyperlipidaemia	103	(38.1)	108	(36.7)	77	(27.4)	90	(33.6)	**0.030**
Smoking	127	(47.0)	125	(42.5)	105	(37.4)	97	(34.5)	**0.014**
Current	72	(26.7)	60	(20.4)	48	(17.1)	44	(15.7)	**0.006**
Former	55	(20.4)	65	(22.1)	57	(20.3)	53	(18.9)	0.816
Family history	62	(23.0)	28	(9.5)	24	(8.5)	18	(6.4)	**0.001**
**Comorbidities at index hospitalization**, n (%)									
Unstable angina	35	(13.0)	29	(9.9)	14	(5.0)	8	(2.8)	**0.003**
STEMI	31	(11.5)	15	(5.1)	14	(5.0)	22	(7.8)	**0.010**
NSTEMI	47	(17.4)	68	(23.1)	49	(17.4)	38	(13.5)	**0.027**
Acute decompensated heart failure	32	(11.9)	69	(23.5)	115	(40.9)	103	(36.7)	**0.001**
Cardiogenic shock	1	(0.4)	7	(2.4)	5	(1.8)	20	(7.1)	**0.001**
Atrial fibrillation	107	(39.6)	122	(41.5)	121	(43.1)	110	(39.1)	0.771
Cardiopulmonary resuscitation	3	(1.1)	4	(1.4)	4	(1.4)	21	(7.5)	**0.001**
Stroke	12	(4.4)	7	(2.4)	18	(6.4)	16	(5.7)	0.111

bpm, beats per minute; CABG, coronary artery bypass grafting; COPD, chronic obstructive pulmonary disease; CRT-D, cardiac resynchronization therapy with defibrillator; DBP, diastolic blood pressure; IQR, interquartile range; mmHg, millimetres of mercury; (N)STEMI, non-ST-segment elevation myocardial infarction; PCI, percutaneous coronary intervention; Q, Quartile; SBP, systolic blood pressure; (s-)ICD, (subcutaneous) implantable cardioverter defibrillator.

Level of significance p ≤ 0.05. Bold type indicates statistical significance.

**Table 2:** Heart-failure related and procedural data.

	**Q1** (*n* = 270)		**Q2** (*n* = 294)		**Q3** (*n* = 281)		**Q4** (*n* = 281)		**p value**
**Heart failure etiology**, n (%)									
Ischemic cardiomyopathy	168	(62.2)	204	(69.4)	183	(65.1)	156	(55.5)	**0.006**
Non-ischemic cardiomyopathy	27	(10.0)	16	(5.4)	17	(6.0)	14	(5.0)	0.070
Hypertensive cardiomyopathy	12	(4.4)	16	(5.4)	16	(5.7)	15	(5.3)	0.921
Congenital heart disease	0	(0.0)	1	(0.3)	0	(0.0)	1	(0.4)	0.589
Valvular heart disease	13	(4.8)	9	(3.1)	24	(8.5)	10	(3.6)	**0.011**
Tachycardia-associated	19	(7.0)	20	(6.8)	17	(6.0)	20	(7.1)	0.957
Tachymyopathy	8	(3.0)	6	(2.0)	6	(2.1)	8	(2.8)	0.853
Pacemaker-induced cardiomyopathy	2	(0.7)	2	(0.7)	1	(04)	4	(1.4)	0.544
Unknown	29	(10.7)	26	(8.8)	23	(8.2)	61	(21.7)	**0.001**
**NYHA functional class,** n (%)									
I/II	214	(79.2)	194	(66.0)	159	(56.6)	163	(58.0)	**0.001**
III	38	(14.1)	70	(23.8)	85	(30.2)	70	(24.9)	
IV	18	(6.7)	30	(10.2)	37	(13.2)	48	(17.1)	
**Echocardiographic data**									
LVEF, %, median (IQR)	45 (45–47)		45 (45–47)		45 (44–47)		45 (45–48)		**0.023**
IVSd, mm, median (IQR)	12 (10–13)		12 (11–13)		12 (11–13)		12 (10–13)		0.061
LVEDD, mm, median (IQR)	49 (45–54)		49 (45–54)		49 (45–55)		48 (44–53)		0.349
TAPSE, mm, median (IQR)	20 (18–23)		20 (18–23)		20 (17–22)		19 (16–22)		**0.025**
LA diameter, mm, median (IQR)	39 (36–44)		41 (36–46)		42 (38–48)		42 (37–47)		**0.007**
LA area, cm^2^, median (IQR)	20 (16–24)		23 (17–26)		24 (18–28)		21 (17–25)		**0.004**
E/A, median (IQR)	0.8 (0.6–1.2)		0.8 (0.6–1.1)		0.9 (0.7–1.3)		0.9 (0.6–1.3)		0.126
E/E, median (IQR)	7.5 (4.0–10.5)		9.5 (6.3–13.0)		11.0 (7.1–16.1)		9.3 (6.5–14.4)		**0.001**
VCI	17 (14–20)		20 (15–25)		22 (15–28)		19 (15–26)		0.084
Diastolic dysfunction, n (%)	190	(70.4)	232	(78.9)	218	(77.6)	189	(67.3)	**0.003**
Moderate/severe aortic stenosis, n (%)	11	(4.1)	21	(7.1)	38	(13.5)	34	(12.1)	**0.001**
Moderate/severe aortic regurgitation, n (%)	7	(2.6)	11	(3.7)	15	(5.3)	13	(4.6)	0.398
Moderate/severe mitral regurgitation, n (%)	18	(6.7)	39	(13.3)	41	(14.6)	36	(12.8)	**0.021**
Moderate/severe tricuspid regurgitation, n (%)	18	(6.7)	43	(14.6)	58	(20.6)	55	(19.6)	**0.001**
**Coronary angiography,** n (%)	176	(65.2)	176	(59.9)	130	(46.3)	99	(35.2)	**0.001**
No evidence of coronary artery disease	46	(26.1)	19	(10.8)	31	(23.8)	19	(19.2)	**0.047**
1-vessel disease	31	(17.6)	35	(19.9)	24	(18.5)	21	(21.2)	
2-vessel disease	37	(21.0)	38	(21.6)	21	(16.2)	18	(18.2)	
3-vessel disease	62	(35.2)	84	(47.7)	54	(41.5)	41	(41.4)	
CABG	6	(3.4)	8	(4.5)	4	(3.1)	5	(5.1)	0.831
Chronic total occlusion	18	(10.2)	24	(13.6)	14	(10.8)	15	(15.2)	0.566
PCI, n (%)	95	(54.0)	93	(52.8)	65	(50.0)	61	(61.6)	0.355
Sent to CABG, n (%)	9	(5.1)	8	(4.5)	7	(5.4)	5	(5.1)	0.989
**Baseline laboratory values**, median (IQR)									
D-dimers, µg/mL	0.27 (0.19–0.35)		0.61 (0.50–0.73)		1.30 (1.09–1.66)		4.10 (3.11–7.53)		**0.129**
Potassium, mmol/L	3.9 (3.7–4.2)		3.9 (3.6–4.2)		3.9 (3.5–4.2)		3.8 (3.5–4.1)		0.129
Sodium, mmol/L	139 (138–141)		139 (138–141)		139 (137–141)		139 (137–141)		0.533
Creatinine, mg/dL	1.0 (0.9–1.2)		1.1 (0.9–1.4)		1.2 (0.9–1.7)		1.1 (0.9–1.6)		**0.001**
eGFR, mL/min/1.73 m^2^	75 (59–93)		62 (47–83)		53 (36–76)		59 (39–84)		**0.001**
Hemoglobin, g/dL	14.0 (12.6–15.1)		13.0 (11.4–14.2)		11.7 (10.5–13.2)		11.7 (10.1–13.2)		**0.001**
WBC count, x 10^9^/L	8.30 (6.44–9.71)		7.93 (6.35–9.58)		8.21 (6.64–10.38)		8.48 (6.36–10.96)		0.170
Platelet count, x 10^9^/L	222 (193–269)		228 (181–274)		235 (171–286)		234 (173–314)		0.419
HbA1c, %	5.9 (5.4–6.7)		6.2 (5.6–7.4)		5.9 (5.4–6.6)		5.9 (5.4–6.8)		**0.031**
LDL- cholesterol, mg/dL	108 (81–133)		93 (73–127)		99 (70–123)		85 (64–120)		**0.002**
HDL- cholesterol, mgl/dL	42 (35–52)		42 (35–50)		42 (35–57)		40 (32–50)		0.240
C-reactive protein, mg/L	3 (3–13)		8 (3–32)		16 (6–48)		33 (10–75)		**0.001**
NT-proBNP, pg/mL	1011 (441–2682)		2195 (967–4381)		4792 (1936–11047)		3420 (1645–9682)		**0.001**
NT-proBNP (eGFR corrected), pg/mL	1062 (365–1945)		1276 (578–2564)		2275 (976–4642)		2168 (1087–4357)		**0.001**
Cardiac troponin I, µg/L	0.02 (0.02–0.15)		0.04 (0.02–0.18)		0.03 (0.02–0.13)		0.05 (0.02–0.28)		**0.004**
**Medication at discharge**, n (%)									
ACE-inhibitor	156	(57.8)	163	(55.8)	132	(48.7)	134	(51.7)	0.142
ARB	67	(24.8)	71	(24.3)	70	(25.8)	58	(22.4)	0.827
Beta-blocker	231	(85.6)	247	(84.6)	219	(80.8)	200	(77.2)	**0.047**
MRA	40	(14.8)	53	(18.2)	45	(16.6)	35	(13.5)	0.466
ARNI	2	(0.7)	6	(2.1)	3	(1.1)	4	(1.5)	0.573
SGLT2-inhibitor	10	(3.7)	20	(6.8)	8	(3.0)	11	(4.2)	0.126
Loop diuretics	91	(33.7)	153	(52.4)	176	(64.9)	147	(56.8)	**0.001**
Statin	193	(71.5)	219	(75.0)	190	(70.1)	161	(62.2)	**0.010**
Digitalis	16	(5.9)	9	(3.1)	11	(4.1)	14	(5.4)	0.363
Amiodarone	8	(3.0)	13	(4.5)	8	(3.0)	5	(1.9)	0.395
ASA	135	(50.0)	165	(56.5)	139	(51.3)	124	(47.9)	0.209
P2Y12-inhibitor	105	(38.9)	136	(46.6)	95	(35.1)	66	(25.5)	**0.001**
DOAC	90	(33.3)	110	(37.7)	100	(36.9)	90	(34.7)	0.700
Vitamin k antagonist	34	(12.6)	19	(6.5)	11	(4.1)	10	(3.9)	**0.001**

Q, Quartile; NYHA, New York Heart Association; LVEF, left ventricular ejection fraction; IQR, interquartile range; IVSd, Interventricular septal end diastole; mm, millimeter; LVEDD, Left ventricular end-diastolic diameter; TAPSE, tricuspid annular plane systolic excursion; LA, left atrial; VCI, vena cava inferior; CABG, coronary artery bypass grafting; PCI, percutaneous coronary intervention; eGFR, estimated glomerular filtration rate; WBC, white blood cells; HbA1c, glycated hemoglobin; LDL, low-density lipoprotein; HDL, high-density lipoprotein; NT-proBNP, aminoterminal pro-B-type natriuretic peptide; ACE, angiotensin converting enzyme; ARB, Angiotensin II Receptor Blockers; MRA, mineralocorticoid receptor antagonist; ARNI, Angiotensin-receptor-neprilysin-inhibitor; SGLT2, Sodium glucose linked transporter 2; ASA, acetylsalicylic acid; DOAC, directly acting oral anticoagulant.

Level of significance p ≤ 0.05. Bold type indicates statistical significance.

Regarding HF-related and procedural data, patients in Q1 more often had ischemic cardiomyopathy (62.2% in Q1 vs. 55.5% in Q4, p = 0.006) and a lower New York Heart Association (NYHA) functional class (I/II 79.2% in Q1 vs. 58.0% in Q4, p = 0.001) with higher rates of prescribed beta-blockers (85.6% in Q1 vs. 77.2% in Q4, p = 0.047), statins (71.5% in Q1 vs. 62.2% in Q4, p = 0.010), P2Y12-inhibitors (38.9% in Q1 vs. 25.5% in Q4, p = 0.001) and lower rates of loop diuretics (33.7% in Q1 vs. 56.8% in Q4, p = 0.001) on discharge. The echocardiographic data showed a significant larger left atrial (LA) diameter (median 42 mm in Q4 vs. 39 mm in Q1, p = 0.007) with higher E/E’ ratio (median 9.3 in Q4 vs. 7.5 in Q1, p = 0.001) and higher prevalence of moderate/severe aortic stenosis (12.1% in Q4 vs. 4.1% in Q1, p = 0.001), mitral regurgitation (12.8% in Q4 vs. 6.7% in Q1, p = 0.021) and tricuspid regurgitation (19.6% in Q4 vs. 6.7% in Q1, p = 0.001) in the upper quartiles. With regard to the other laboratory values, the estimated glomerular fraction rate (eGFR) (median 59 mL/min/1.73 m^2^ in Q4 vs. 75 mL/min/1.73 m^2^ in Q1, p = 0.001), the hemoglobin (median 11.7 g/dl in Q4 vs. 14.0 g/dl in Q1, p = 0.001) and the LDL-cholesterol (median 85 mg/dL in Q4 vs. 108 mg/dL in Q1, p = 0.002) levels were lower in the upper quartiles, while the CRP (median 33 mg/L in Q4 vs. 3 mg/L in Q1, p = 0.001) and the eGFR corrected NT-proBNP (median 2168 pg/mL in Q4 vs. 1062 pg/mL in Q1, p = 0.001) levels showed significant higher values (Table 2).

### 3.2 Correlations of D-dimer levels with echocardiographic and laboratory data

In patients with HFmrEF, the D-dimer levels correlated with age (*r* = 0.271; p = 0.001), creatinine (*r* = 0.131; p = 0.001), CRP (*r* = 0.421; p = 0.001), NT-proBNP (*r* = 0.298; p = 0.001) and cardiac troponin I (*r* = 0.120; p = 0.001), while negative correlations were observed in body mass index (*r* = -0.155; p = 0.001), tricuspid annular plane systolic excursion (TAPSE) (*r* = -0.099; p = 0.001), eGFR (*r* = -0.192; p = 0.001), and hemoglobin (*r* = -0.410; p = 0.001) (Table 3).

**Table 3:** Correlations of D-dimers with laboratory and clinical parameters.

	D-dimers	
	r	p value
Age (years)	0.271	**0.001**
Body mass index (kg/m^2^)	−0.155	**0.001**
LVEF (%)	0.012	0.689
IVSd (mm)	−0.005	0.863
LVEDD (mm)	−0.045	0.171
TAPSE (mm)	−0.099	**0.001**
Creatinine (mg/dL)	0.131	**0.001**
eGFR (ml/min/1.73 m^2^)	−0.192	**0.001**
Hemoglobin (g/dL)	−0.410	**0.001**
C-reactive protein (mg/L)	0.421	**0.001**
NT-proBNP (pg/mL)	0.298	**0.001**
Cardiac troponin I (µg/L)	0.120	**0.001**

LVEF, left ventricular ejection fraction; IVSd, interventricular septum thickness end diastole; mm, millimeter; LVEDD, left ventricular end-diastolic diameter; TAPSE, tricuspid annular plane systolic excursion; eGFR, estimated glomerular filtration rate; NT-proBNP, aminoterminal pro-B-type natriuretic peptide.

Level of significance p ≤ 0.05. Bold type indicates statistical significance.

### 3.3 Prognostic impact of D-dimer levels in patients with HFmrEF

**Fig. 2:**
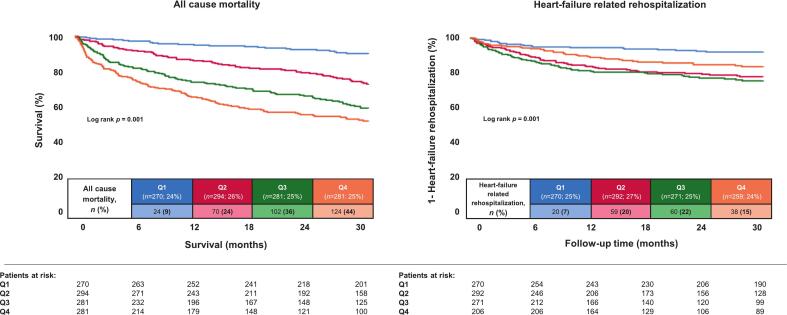
Kaplan-Meier analyses with regard to the risks of all-cause mortality and HF-related rehospitalization within the entire study cohort (analysis 1).

After a median follow-up of 30 months, the risk of all-cause mortality was significantly increased in patients with higher D-dimer levels (Q4 44.1% vs. Q1 8.9%, p = 0.001). Univariate HR were 6.817 (95% CI 4.401–10.561, p = 0.001) for Q4, 5.123 (95% CI 3.283 – 7.994; p = 0.001) for Q3 and 2.986 (95% CI 1.878 – 4.478; p = 0.001) for Q2, compared to Q1 (Table 4**,** Fig. 2). With regard to the key secondary endpoints, patients in the upper quartiles had a higher all-cause already during index hospitalization (Q4 7.8% vs. Q1 0%, p = 0.001), whereas both the risks of cardiac (Q4 3.2% vs. Q1 0%, p = 0.001) and non-cardiac in-hospital mortality were higher (Q4 4.6% vs. Q1 0%, p = 0.001), alongside with higher risks of HF-related rehospitalization at 12 (Q4 9.7% vs. Q1 5.2%, p = 0.001) and at 30 months (Q4 14.7% vs. Q1 7.4%, p = 0.001), all-cause mortality at 12 months (Q4 33.1% vs. Q1 4.4%, p = 0.001) and MACCE at 30 months (Q4 49.5% vs. Q1 21.9%, p = 0.001). Univariate HR for HF-related rehospitalization were 2.523 (95% CI 1.467 – 4.339: p = 0.001) for Q4, 3.820 (95% CI 2.301 – 6.341; p = 0.001) for Q3 and 3.208 (95% CI 1.931 – 5.329; p = 0.001) for Q2, compared to Q1 (Fig. 2).

**Table 4:** Follow-up data, primary and secondary endpoints.

	**Q1** (*n* = 270)		**Q2** (*n* = 294)		**Q3** (*n* = 281)		**Q4** (*n* = 281)		**p value**
**Primary endpoint**, n (%)									
All-cause mortality, at 30 months	24	(8.9)	70	(23.8)	102	(36.3)	124	(44.1)	**0.001**
**Secondary endpoints**, n (%)									
All-cause mortality, in-hospital	0	(0.0)	2	(0.7)	10	(3.6)	22	(7.8)	**0.001**
Cardiac mortality, in-hospital	0	(0.0)	0	(0.0)	6	(2.1)	9	(3.2)	**0.001**
Non-cardiac mortality, in-hospital	0	(0.0)	2	(0.7)	4	(1.4)	13	(4.6)	**0.001**
All-cause mortality, at 12 months	12	(4.4)	39	(13.3)	70	(24.9)	93	(33.1)	**0.001**
Heart failure-related rehospitalization, at 30 months	20	(7.4)	59	(20.2)	60	(22.1)	38	(14.7)	**0.001**
Heart failure-related rehospitalization, at 12 months	14	(5.2)	45	(15.4)	48	(17.7)	25	(9.7)	**0.001**
Cardiac rehospitalization, at 30 months	66	(24.4)	88	(30.1)	79	(29.2)	63	(24.3)	0.271
Coronary revascularization, at 30 months	31	(11.5)	28	(9.6)	19	(7.0)	15	(5.8)	0.081
Acute myocardial infarction, at 30 months	9	(3.3)	14	(4.8)	13	(4.8)	10	(3.9)	0.784
Stroke, at 30 months	9	(3.3)	9	(3.1)	7	(2.6)	10	(3.9)	0.867
MACCE, at 30 months	59	(21.9)	100	(34.0)	123	(43.8)	139	(49.5)	**0.001**
**Follow-up data**, median (IQR)									
Hospitalization time, days	6 (4–9)		7 (5–11)		10 (7–17)		12 (7–20)		**0.001**
ICU time, days	0 (0–1)		0 (0–1)		0 (0–1)		0 (0–2)		**0.018**
Follow-up time, days	1500 (849–2077)		976 (474–1778)		772 (301–1371)		591 (197–11353)		**0.001**

Q, Quartile; MACCE, major adverse cardiac and cerebrovascular events; IQR, Interquartile ratio; ICU, intensive care unit.

Level of significance p ≤ 0.05. Bold type indicates statistical significance.

Further secondary endpoints such as cardiac rehospitalization, coronary revascularization, AMI or stroke, all at 30 months follow-up, did not differ between the quartiles (Table 4).

After multivariable adjustment, higher D-dimer levels (i.e., Q2 (HR = 1.953; 95% CI 1.133–3.366, p = 0.016), Q3 (HR = 2.051; 95% CI 1.195–3.519, p = 0.009) and Q4 (HR = 3.228; 95% CI 1.901–5.479, p = 0.001), compared to Q1 as reference group), as well as age (HR = 1.031; 95% CI 1.017–1.044, p = 0.001, per year increase), prior congestive HF (HR = 1.323; 95% CI 1.011–1.732, p = 0.042), malignancies (HR = 1.950; 95% CI 1.429–2.660, p = 0.001), acute decompensated HF (HR = 1.662; 95% CI 1.219–2.265, p = 0.001), atrial fibrillation (HR = 1.460; 95% CI 1.022–2.085, p = 0.037) as well as hemoglobin (HR = 0.883; 95% CI 0.829–0.940, p = 0.001) were identified as independent risk factors for all-cause mortality at 30 months.

Regarding the risk of HF-related rehospitalization, we found Q2 (HR = 2.742; 95% CI 1.508–4.985, p = 0.001) and Q3 (HR = 2.753; 95% CI 1.478–5.129, p = 0.001), again compared to Q1 as reference group, as well as prior chronic HF (HR = 1.550; 95% CI 1.082–2.220, p = 0.017), acute decompensated HF (HR = 1.542; 95% CI 1.021–2.330, p = 0.040), atrial fibrillation (HR = 1.829; 95% CI 1.104–3.031, p = 0.019) and NYHA functional class (HR = 1.319; 95% CI 1.081–1.609, p = 0.006) to be independent risk factors (Table 5),

**Table 5:** Multivariate Cox regression analyses with regard to all-cause mortality and heart-failure related rehospitalization at 30 months.

	All-cause mortality at 30 months			Heart failure related rehospitalization		
	HR	95% CI	p value	HR	95% CI	p value
Age	1.031	1.017–1.044	**0.001**	1.005	0.988–1.023	0.568
Sex	1.263	0.969–1.646	0.084	0.933	0.657–1.327	0.701
Prior chronic HF	1.323	1.011–1.732	**0.042**	1.550	1.082–2.220	**0.017**
BMI (per kg/m^2^ increase)	0.953	0.927–0.980	**0.001**	1.024	0.992–1.056	0.139
Malignancies	1.950	1.429–2.660	**0.001**	0.782	0.417–1.465	0.442
Diabetes mellitus	1.033	0.792–1.347	0.811	0.978	0.681–1.403	0.902
Acute myocardial infarction	1.264	0.887–1.800	0.194	1.037	0.633–1.698	0.885
Acute decompensated HF	1.662	1.219–2.265	**0.001**	1.542	1.021–2.330	**0.040**
Atrial fibrillation	1.460	1.022–2.085	**0.037**	1.829	1.104–3.031	**0.019**
Anticoagulation	0.892	0.623–1.276	0.531	1.563	0.917–2.664	0.101
Ischemic cardiomyopathy	0.708	0.529–0.948	**0.021**	1.069	0.728–1.572	0.733
NYHA functional class	1.017	0.881–1.172	0.822	1.319	1.081–1.609	**0.006**
eGFR (per ml/min/1.73 m^2^ increase)	0.996	0.991–1.001	0.095	0.994	0.987–1.001	0.084
Hemoglobin (per g/dl increase)	0.883	0.829–0.940	**0.001**	0.931	0.855–1.013	0.095
C-reactive protein	1.001	0.999–1.003	0.180	0.997	0.993–1.001	0.174
2nd D-dimer quartile	1.953	1.133–3.366	**0.016**	2.742	1.508–4.985	**0.001**
3rd D-dimer quartile	2.051	1.195–3.519	**0.009**	2.753	1.478–5.129	**0.001**
4th D-dimer quartile	3.228	1.901–5.479	**0.001**	1.884	0.978–3.629	0.058
1st D-dimer quartile	reference group			reference group		

HR, hazard ratio; CI, confidence interval; HF, heart failure; BMI, body mass index; NYHA, New York Heart Association; eGFR, estimated glomerular filtration rate.

Level of significance p ≤ 0.05. Bold type indicates statistical significance.

An independent association of D-dimer levels and the risk of all-cause mortality at 30 months was observed in patients on anticoagulation (Q3 HR = 1.926; 95% CI 1.014–3.656, p = 0.045; Q4; HR = 2.851; 95% CI 1.529–5.318, p = 0.001, Q1: reference group) and without anticoagulation (Q3: HR = 2.777; 95% CI 0.915–8.429, p = 0.071; Q4: HR = 4.632; 95% CI 1.549–13.858, p = 0.006; Q1: reference group) after multivariable adjustments.

### 3.4 Prognostic impact of D-dimer levels in patients without conditions associated with increased D-dimer levels

**Fig. 3:**
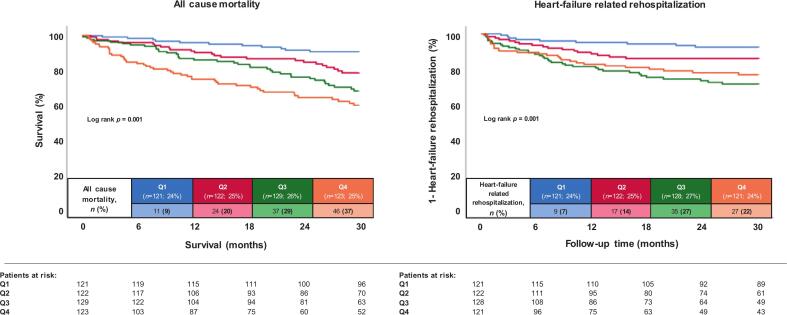
Kaplan-Meier analyses with regard to the risks of all-cause mortality and HF-related rehospitalization after excluding patients with conditions associated with increased D-dimer levels (analysis 2).

For analysis 2, 631 patients with other conditions associated with elevated D-dimers including 284 patients due to AMI, 268 due to infection, 131 due to malignancy, 53 due to stroke, 51 due to PE, 33 due to cardiogenic shock, 32 due to CPR and 25 due to VTE were excluded, whereby a patient could meet multiple above mentioned excluding criteria. The final cohort for analysis 2 comprised 495 patients (Fig. 1). Baseline characteristics were mostly comparable to analysis 1 (Supplemental tables 1 and 2). The risk of all-cause mortality was still increased in patients with higher D-dimer levels (Q4 37.4% vs. Q1 9.1%, p = 0.001) (Fig. 3**,** Supplemental table 3). Univariate HR were 5.508 (95% CI 2.851–10.643, p = 0.001) for Q4, 3.733 (95% CI 1.903 – 7.320; p = 0.001) for Q3 and 2.408 (95% CI 1.179 – 4.916; p = 0.016) for Q2, compared to Q1. After multivariable adjustment, higher D-dimer levels (i.e., Q3 (HR = 2.442; 95% CI 1.071–5.564, p = 0.034) and Q4 (HR = 2.880; 95% CI 1.280–6.482, p = 0.011), compared to Q1 as reference group) were still identified as independent risk factors for all-cause mortality at 30 months (Supplemental table 4**)**.

## 4 Discussion

The aim of the present study was to investigate the prognostic impact of D-dimer levels in consecutive patients hospitalized with HFmrEF. Elevated D-dimer levels were more common in older patients with increased burden of cardiac comorbidities. They were associated with a higher risk of all-cause mortality and HF-related rehospitalization, even after multivariable adjustments, suggesting an independent prognostic impact of D-dimer levels in patients hospitalized with HFmrEF. Consistent findings were demonstrated even after excluding patients with conditions associated with elevated D-dimer levels and irrespective of treatment with anticoagulation. D-dimer levels may serve as an independent prognostic risk factor, even in patients with HFmrEF.

The prognostic impact of elevated D-dimers in HF has been investigated within various studies, however, data investigating D-dimer levels in patients with HFmrEF remain scarce. A subgroup analysis of the COMMANDER-HF study, investigating 4107 patients with HFrEF and coronary artery disease, found higher D-dimer levels, even above 0.256 µg/mL, as an independent risk factor for all-cause death, HF-related rehospitalization and embolic stroke [20]. The lower cut-off in this study may be related to the much bigger cohort of HFrEF patients, who are over-all more at risk than HFmrEF patients and the distribution in tertials, not quartiles. Another observational study including 1355 patients above 60 years with chronic HF suggested elevated D-dimer levels > 0.885 µg/mL as an independent risk factor for all-cause death for patients with preserved (LVEF > 50%) and reduced LVEF (<50%). It should be noted, however, that there was no further differentiation made with regard to the cause of death and HFmrEF was not specifically investigated [14]. An observational study including 289 HFmrEF patients showed a higher all-cause mortality after 12 months with D-dimer levels > 1.295 µg/mL unadjusted, while adjusted for age, creatinine, diabetes, among other factors it was no longer significant [21], contributing to overall heterogeneous findings regarding the impact of D-dimer levels in HF patients. To our best knowledge, there is no study investigating the association of D-dimer levels in HFmrEF patients since the new definition from 2016. With a cohort of more than 1000 patients, allowing a representative subgroup analysis of patients with D-dimers only raised by HF, and a 2.5-year follow-up, this study adds meaningful evidence about D-dimer influence on HFmrEF. Furthermore, the association of D-dimer levels and adverse prognosis was demonstrated within all patients (all-comers setting) and even after excluding patients with conditions associated with an increase of D-dimer levels and irrespective of treatment with anticoagulants.

The reason of elevated D-dimer being a risk factor for poor outcome is not exactly known today. First of all D-dimers reflect fibrinolysis and a hypercoagulable state, which is associated with inflammation by proinflammatory cytokines and activation of extrinsic coagulation cascade [22]. Proinflammatory cytokines are released as a reaction of the hemodynamic stress in HF, activating the coagulation system [23]. Furthermore, D-dimers themselves have been demonstrated to induce cytokine release in monocytes, thereby intensifying inflammation and contributing to the pathogenesis of HF, although anti-inflammatory therapy was not successful in HF so far [23], [24]. They are also discussed as being a general marker for increased inflammatory activity due to HF, which would be reflected in the higher rates of HF-related rehospitalization, because progressing HF leads to more frequently rehospitalizations [12], [25], [26]. It may also indicate a clinical unrecognized prothrombotic condition like unknown cancer or paroxysmal atrial fibrillation [18]. In the COMMANDER-HF cohort, elevated D-dimers were significantly associated with a higher rate of ischemic stroke, but not AMI. Because the additional anticoagulation with low-dose rivaroxaban did lower the rates of ischemic stroke in patients with elevated D-dimers, the authors discussed clinically not apparent prothrombotic diseases as the possible reason [20]. Regarding pathophysiological differences in the HF phenotypes, HFpEF is mainly driven by systemic inflammation and fibrosis, leading to higher filling pressures and reduced vascular capacity. This results in higher flow rates and more endothelial dysfunction. Compared with HFpEF, HFrEF is less characterised by inflammation but more by lower flow rates and higher venous capacity and stasis [27]. HFmrEF is associated with systemic inflammation markers similar to HFpEF, but it is still considered as an intermediate state, specifically regarding the higher rates of ischemic cardiomyopathy which may be closely linked to HFrEF [28].

If the poorer outcome of HFmrEF patients with higher D-dimer levels may predominantly be caused by systemic inflammation, venous stasis or the proinflammatory effect of D-dimers themself, creating a circulus vitiosus, remains unclear [23], [27]. The last would explain D-dimers as an independent pathophysiological risk factor, because not HF-related elevation of D-dimers by hypercoagulation, e.g. in DVT, could worsen HF through the D-dimer induced inflammation [24] Elevated D-dimer levels are also influenced by many different other factors, concurrent with the existing literature and the results of the present study. With rising age, D-dimer levels are higher too, presumably by a higher prevalence of inflammatory conditions, which is accepted as physiological and taken into account with adjusted cut-off-values [29], [30]. Despite this physiological elevation, many diseases are associated with higher D-dimer levels, which is also reflected in the results of the present study by significant higher prevalence with higher D-dimer levels. Patients with CKD are associated with higher D-dimer levels due to reduced renal clearance of D-dimers and systemic inflammation as well as hypercoagulability [31], [32]. Atherosclerosis in patients with PAD is characterised by the initiation of coagulation and fibrinolysis, resulting in elevated D-dimer levels [33]. Further the complex changes of coagulation in patients with liver cirrhosis results in elevated D-dimer levels, although not necessarily representing a procoagulant state [34], [35]. Acute decompensated HF results, with rising severity reflected by higher NYHA functional class and NT-proBNP levels in the upper quartiles, in systemic inflammation and hypercoagulable state as well as arterial hypertension and diastolic dysfunction [13], [36], [37]. Anaemia, a common comorbidity among patients with HFmrEF, is independently associated with elevated D-dimer levels, especially in patients with HFmrEF [38], [39]. To rule out the possibility that the positive endpoints are only correlated due to the above-mentioned diseases, we performed a multivariable analysis, which found higher D-dimer levels to be an independent risk factor for all-cause mortality und HF rehospitalization.

Finally elevated D-dimer seem to indicate a poorer outcome in patients with HFmrEF and should emphasise an accurate and effective HF therapy and a critical clinical evaluation of other underlying causes of elevated D-dimers to lower adverse outcomes. Larger prospective cohorts are needed to define the association between elevated D-dimer levels and stroke or AMI risk in HFmrEF and to identify subgroups that might benefit from anticoagulation, as well as evaluating D-dimers in prognostic scores. Biomarkers are commonly used in prognostic scores for HF like the Meta-analysis Global Group in Chronic Heart Failure (MAGGIC)-Score, evaluating mortality in HF. D-dimers, reflecting the coagulation system as a source of inflammation, could be added in future scores for risk stratification [40]. Despite the association between HF, systemic inflammation and hypercoagulation, a more precise understanding in therapy is mandatory for treating HFmrEF more effective in future.

## 5 Study limitations

The retrospective study and single-centre study design need to be acknowledged as a major limitation. Within the present all-comers registry, D-dimer measurement was performed during routine clinical practice and the indication for D-dimer testing was performed according to the discretion of the treating physicians. Therefore, a significant proportion of patients had to be excluded in whom no D-dimer measurement was performed. Patients with D-dimer testing were older and had more advanced HF symptoms, reflected by higher NYHA functional class, with higher rates of concomitant AMI and cardiovascular risk factors (Supplemental table 5 and 6). Therefore, prospective studies are deemed necessary investigating the prognostic role of D-dimer levels in patients with HFmrEF without any pre-selection. Although index echocardiography was performed following hemodynamic stabilisation in patients with AMI or HF-decompensation and most laboratory values were taken at the beginning of hospitalisation, information about treatment initiation was not available in the vast majority of patients. Furthermore, recurrent hospitalizations were only recorded at our institution. For the present study, risk stratification was performed according to the D-dimer at the initial hospitalization and no D-dimer data was recorded during follow-up. NT-proBNP levels were obtained in only 504 patients with d-dimer testing and therefore not included into the main multivariable Cox regression model. However, even when additionally adjusted for NT-proBNP levels, D-dimers were still associated with the risk of 30-months all-cause mortality (Q4 vs. Q1: HR = 2.760, 95% CI 1.219 – 6.248, p = 0.015). Finally, the causes of death beyond index hospitalization were beyond the scope of the study.

## 6 Conclusions

The present study suggests that elevated D-dimer levels represent an independent risk factor for all-cause mortality at 30 months in consecutive patients with HFmrEF.

## 7 Ethical standard statements

The registry was carried out according to the principles of the declaration of Helsinki and was approved by the medical ethics committee II of the Medical Faculty Mannheim, University of Heidelberg, Germany (ethical approval code: 2022–818).

## CRediT authorship contribution statement

**Finn Kronberg:** Writing – original draft, Software, Methodology, Investigation, Data curation. **Tobias Schupp:** Writing – review & editing, Visualization, Validation, Supervision, Software, Resources, Project administration, Methodology, Investigation, Formal analysis, Data curation, Conceptualization. **Michael Behnes:** Visualization, Validation, Resources, Project administration, Conceptualization. **Michelle Goertz:** Data curation. **Marielen Reinhardt:** Data curation. **Noah Abel:** Data curation. **Alexander Schmitt:** Data curation. **Felix Lau:** Software, Formal analysis. **Mohammad Abumayyaleh:** Methodology. **Thomas Bertsch:** Validation. **Ibrahim Akin:** Project administration, Conceptualization. **Kathrin Weidner:** Writing – review & editing, Validation, Supervision, Resources, Investigation, Data curation.

## Funding

This manuscript did not receive any funding.

## Declaration of competing interest

The authors declare that they have no known competing financial interests or personal relationships that could have appeared to influence the work reported in this paper.
