# Supplementary material for: D-dimer levels and outcomes in heart failure with mildly reduced ejection fraction
Source: Int J Cardiol Heart Vasc. 2026 Mar 29;64:101915. doi: 10.1016/j.ijcha.2026.101915 (PMC13062528; doi:10.1016/j.ijcha.2026.101915)
Supplement: Supplementary Data 1 [file mmc1.docx]

| **Supplemental Table 1. Baseline characteristics of patients included in analysis 2.** | | | | | | | | | |  |
| --- | --- | --- | --- | --- | --- | --- | --- | --- | --- | --- |
|  | **Q1**  (*n*=121) | | **Q2**  (*n*=122) | | **Q3**  (*n*=129) | | **Q4**  (*n*=123) | | **p value** |  |
| **Age**, median (IQR) | 65 (55-77) | | 76 (65-82) | | 76 (66-83) | | 78 (79-85) | | **0.001** | |
| **Male sex**, n (%) | 80 | (66.1) | 82 | (67.2) | 88 | (68.2) | 72 | (58.5) | 0.365 | |
| **Body mass index,** kg/m^2^, median (IQR) | 28.2 (24.7-31.9) | | 27.7 (25.1-31.4) | | 26.9 (24.6-31.8) | | 27.1 (23.1-30.9) | | 0.201 | |
| **SBP**, mmHg, median (IQR) | 140 (130-157) | | 147 (131-165) | | 144 (126-167) | | 148 (130-164) | | 0.200 |  |
| **DBP**, mmHg, median (IQR) | 81 (72-89) | | 81 (73-91) | | 77 (68-90) | | 79 (70-92) | | 0.212 | |
| **Heart rate**, bpm, median (IQR) | 80 (70-97) | | 83 (70-102) | | 79 (66-93) | | 84 (69-100) | | 0.404 | |
| **Medical history**, n (%) |  |  |  |  |  |  |  |  |  | |
| Coronary artery disease | 51 | (42.1) | 61 | (50.0) | 72 | (55.8) | 51 | (41.5) | 0.070 | |
| Prior myocardial infarction | 28 | (23.1) | 37 | (30.3) | 38 | (29.5) | 36 | (29.3) | 0.578 | |
| Prior PCI | 36 | (29.8) | 49 | (40.2) | 53 | (41.1) | 37 | (30.1) | 0.100 | |
| Prior CABG | 10 | (8.3) | 11 | (9.0) | 19 | (14.7) | 15 | (12.2) | 0.335 | |
| Prior valvular surgery | 10 | (8.3) | 8 | (6.6) | 7 | (5.4) | 8 | (6.5) | 0.844 | |
| Congestive heart failure | 53 | (43.8) | 58 | (47.5) | 54 | (41.9) | 49 | (39.8) | 0.656 | |
| Decompensated heart failure <12 months | 15 | (12.4) | 19 | (15.6) | 16 | (12.4) | 24 | (19.5) | 0.344 | |
| Prior ICD | 5 | (4.1) | 4 | (3.3) | 2 | (1.6) | 0 | (0.0) | 0.126 | |
| Prior sICD | 2 | (1.7) | 1 | (0.8) | 3 | (2.3) | 0 | (0.0) | 0.362 | |
| Prior CRT-D | 6 | (5.0) | 0 | (0.0) | 5 | (3.9) | 2 | (1.6) | 0.068 | |
| Prior Pacemaker | 11 | (9.1) | 13 | (10.7) | 15 | (11.6) | 15 | (12.2) | 0.874 | |
| Chronic kidney disease | 20 | (16.5) | 40 | (32.8) | 58 | (45.0) | 58 | (47.2) | **0.001** | |
| Peripheral artery disease | 0 | (0.0) | 8 | (6.6) | 13 | (10.1) | 17 | (13.8) | **0.001** | |
| Stroke | 10 | (8.3) | 15 | (12.3) | 17 | (13.2) | 21 | (17.1) | 0.230 | |
| Liver cirrhosis | 1 | (0.8) | 1 | (0.8) | 2 | (1.6) | 7 | (5.7) | **0.026** | |
| Malignancy | 0 | (0.0) | 0 | (0.0) | 0 | (0.0) | 0 | (0.0) | - | |
| COPD | 12 | (9.9) | 12 | (9.9) | 22 | (17.1) | 24 | (19.5) | 0.058 | |
| **Cardiovascular risk factors,** n (%) |  |  |  |  |  |  |  |  |  | |
| Arterial hypertension | 88 | (72.7) | 97 | (79.5) | 105 | (81.4) | 108 | (87.8) | **0.030** | |
| Diabetes mellitus | 35 | (28.9) | 48 | (39.3) | 50 | (38.8) | 41 | (33.3) | 0.271 | |
| Hyperlipidaemia | 50 | (41.3) | 47 | (38.5) | 38 | (29.5) | 40 | (32.5) | 0.186 | |
| Smoking | 50 | (41.3) | 45 | (36.9) | 49 | (38.0) | 50 | (40.7) | 0.875 | |
| Current | 23 | (19.0) | 25 | (20.5) | 21 | (16.3) | 16 | (13.0) | 0.423 | |
| Former | 27 | (22.3) | 20 | (16.4) | 28 | (21.7) | 34 | (27.6) | 0.210 | |
| Family history | 21 | (17.4) | 15 | (12.3) | 11 | (8.5) | 13 | (10.6) | 0.175 | |
| **Comorbidities at index hospitalization**,  n (%) |  |  |  |  |  |  |  |  |  | |
| Unstable angina | 24 | (19.8) | 23 | (18.9) | 17 | (13.2) | 6 | (4.9) | **0.003** | |
| STEMI | 0 | (0.0) | 0 | (0.0) | 0 | (0.0) | 0 | (0.0) | - | |
| NSTEMI | 0 | (0.0) | 0 | (0.0) | 0 | (0.0) | 0 | (0.0) | - | |
| Acute decompensated heart failure | 15 | (12.4) | 27 | (22.1) | 45 | (34.9) | 55 | (44.7) | **0.001** | |
| Cardiogenic shock | 0 | (0.0) | 0 | (0.0) | 0 | (0.0) | 0 | (0.0) | - | |
| Atrial fibrillation | 67 | (55.4) | 56 | (45.9) | 55 | (42.6) | 59 | (48.0) | 0.227 | |
| Cardiopulmonary resuscitation | 0 | (0.0) | 0 | (0.0) | 0 | (0.0) | 0 | (0.0) | - | |
| Stroke | 0 | (0.0) | 0 | (0.0) | 0 | (0.0) | 0 | (0.0) | - | |
| Q, Quartile; IQR, interquartile range; SBP, systolic blood pressure; mmHg, millimetres of mercury; DBP, diastolic blood pressure; bpm, beats per minute; PCI, percutaneous coronary intervention; CABG, coronary artery bypass grafting; (s-)ICD, (subcutaneous) implantable cardioverter defibrillator; CRT-D, cardiac resynchronization therapy with defibrillator; COPD, chronic obstructive pulmonary disease; (N)STEMI, non-ST-segment elevation myocardial infarction.  Level of significance p≤0.05. Bold type indicates statistical significance. | | | | | | | | | |  |
