# Supplementary material for: D-dimer levels and outcomes in heart failure with mildly reduced ejection fraction
Source: Int J Cardiol Heart Vasc. 2026 Mar 29;64:101915. doi: 10.1016/j.ijcha.2026.101915 (PMC13062528; doi:10.1016/j.ijcha.2026.101915)
Supplement: Supplementary Data 2 [file mmc2.docx]

| **Supplemental Table 2. Heart failure related and procedural data of patients included in analysis 2.** | | | | | | | | | |
| --- | --- | --- | --- | --- | --- | --- | --- | --- | --- |
|  | **Q1**  (*n*=121) | | **Q2**  (*n*=122) | | **Q3**  (*n*=129) | | **Q4**  (*n*=123) | | **p value** |
| **Heart failure etiology**, n (%) |  |  |  |  |  |  |  |  |  |
| Ischemic cardiomyopathy | 54 | (44.6) | 67 | (54.9) | 77 | (59.7) | 66 | (53.7) | 0.116 |
| Non-ischemic cardiomyopathy | 21 | (17.4) | 6 | (4.9) | 11 | (8.5) | 5 | (4.1) | **0.001** |
| Hypertensive cardiomyopathy | 5 | (4.1) | 7 | (5.7) | 15 | (11.6) | 10 | (8.1) | 0.123 |
| Congenital heart disease | 0 | (0.0) | 1 | (0.8) | 0 | (0.0) | 0 | (0.0) | 0.382 |
| Valvular heart disease | 12 | (9.9) | 6 | (4.9) | 8 | (6.2) | 10 | (8.1) | 0.455 |
| Tachycardia-associated | 10 | (8.3) | 18 | (14.8) | 8 | (6.2) | 11 | (8.9) | 0.121 |
| Tachymyopathy | 6 | (5.0) | 4 | (3.3) | 4 | (3.1) | 4 | (3.3) | 0.848 |
| Pacemaker-induced cardiomyopathy | 1 | (0.8) | 1 | (0.8) | 2 | (1.6) | 1 | (0.8) | 0.917 |
| Unknown | 18 | (14.9) | 16 | (13.1) | 8 | (6.2) | 20 | (16.3) | 0.074 |
| **NYHA functional class,** n (%) |  |  |  |  |  |  |  |  |  |
| I/II | 93 | (76.9) | 81 | (66.4) | 79 | (41.3) | 57 | (46.3) | **0.001** |
| III | 17 | (14.0) | 28 | (23.0) | 36 | (27.9) | 43 | (35.0) |  |
| IV | 11 | (9.1) | 13 | (10.7) | 14 | (10.9) | 23 | (18.7) |  |
| **Echocardiographic data** |  |  |  |  |  |  |  |  |  |
| LVEF, %, median (IQR) | 45 (45-48) | | 45 (45-46) | | 45 (45-46) | | 45 (45-47) | | 0.185 |
| IVSd, mm, median (IQR) | 11 (10-13) | | 12 (11-13) | | 12 (11-14) | | 12 (11-13) | | 0.205 |
| LVEDD, mm, median (IQR) | 50 (45-54) | | 50 (45-54) | | 50 (45-54) | | 50 (45-55) | | 0.846 |
| TAPSE, mm, median (IQR) | 20 (17-23) | | 20 (18-24) | | 20 (17-22) | | 19 (17-22) | | 0.437 |
| LA diameter, mm, median (IQR) | 38 (34-45) | | 40 (36-46) | | 44 (40-49) | | 46 (40-51) | | **0.001** |
| LA area, cm^2^, median (IQR) | 23 (16-27) | | 22 (18-26) | | 24 (18-27) | | 25 (19-29) | | 0.211 |
| E/A, median (IQR) | 0.8 (0.6-1.1) | | 0.7 (0.6-0.9) | | 0.9 (0.6-1.4) | | 0.9 (0.7-1.4) | | 0.080 |
| E/E`, median (IQR) | 6.9 (4.5-9.5) | | 9.0 (5.0-12.3) | | 9.0 (6.0-15.4) | | 11.0 (6.1-16.3) | | **0.012** |
| VCI | 17 (15-23) | | 19 (16-28) | | 22 (17-28) | | 21 (17-28) | | 0.170 |
| Diastolic dysfunction, n (%) | 83 | (68.6) | 100 | (82.0) | 103 | (79.8) | 83 | (67.5) | **0.012** |
| Moderate-severe aortic stenosis, n (%) | 6 | (5.0) | 4 | (3.3) | 13 | (10.1) | 18 | (14.6) | **0.005** |
| Moderate-severe aortic regurgitation, n (%) | 4 | (3.3) | 1 | (0.8) | 7 | (5.4) | 5 | (4.1) | 0.239 |
| Moderate-severe mitral regurgitation, n (%) | 8 | (6.6) | 15 | (12.3) | 16 | (12.4) | 18 | (14.6) | 0.240 |
| Moderate-severe tricuspid regurgitation, n (%) | 8 | (6.6) | 16 | (13.1) | 24 | (18.6) | 26 | (21.1) | **0.007** |
| **Coronary angiography,** n (%) | 67 | (55.4) | 66 | (54.1) | 54 | (41.9) | 51 | (41.5) | **0.037** |
| No evidence of coronary artery disease | 31 | (46.3) | 20 | (30.3) | 14 | (25.9) | 18 | (35.3) | 0.090 |
| 1-vessel disease | 10 | (14.9) | 8 | (12.1) | 8 | (14.8) | 11 | (21.6) |  |
| 2-vessel disease | 7 | (10.4) | 17 | (25.8) | 7 | (13.0) | 7 | (13.7) |  |
| 3-vessel disease | 19 | (28.4) | 21 | (31.8) | 25 | (46.3) | 15 | (29.4) |  |
| CABG | 5 | (7.5) | 1 | (1.5) | 3 | (5.6) | 2 | (3.9) | 0.418 |
| Chronic total occlusion | 6 | (9.0) | 8 | (12.1) | 4 | (7.4) | 7 | (13.7) | 0.690 |
| PCI, n (%) | 18 | (26.9) | 19 | (28.8) | 19 | (35.2) | 18 | (35.3) | 0.667 |
| Sent to CABG, n (%) | 3 | (4.5) | 1 | (1.5) | 2 | (3.7) | 2 | (3.9) | 0.797 |
| **Baseline laboratory values**, median (IQR) |  |  |  |  |  |  |  |  |  |
| D-dimers, µg/mL | 0.22 (0.19-0.29) | | 0.47 (0.40-0.54) | | 0.84 (0.73-1.10) | | 2.49 (1.62-3.86) | | **0.001** |
| Potassium, mmol/L | 4.0 (3.6-4.2) | | 3.9 (3.6-4-2) | | 3.9 (3.6-4.2) | | 3.9 (3.6-4.3) | | 0.944 |
| Sodium, mmol/L | 140 (138-141) | | 139 (138-141) | | 139 (138-141) | | 139 (138-142) | | 0.621 |
| Creatinine, mg/dL | 1.0 (0.9-1.2) | | 1.1 (0.9-1.5) | | 1.2 (1.0-1.5) | | 1.2 (1.0-1.6) | | **0.001** |
| eGFR, mL/min/1.73 m^2^ | 72 (58-93) | | 64 (47-82) | | 59 (42-78) | | 52 (33-72) | | **0.001** |
| Hemoglobin, g/dL | 14.0 (12.8-15.1) | | 13.3 (12.3-14.7) | | 12.9 (11.5-14.2) | | 11.9 (10.3-13.3) | | **0.001** |
| WBC count, x 10^9^/L | 7.73 (6.29-9.69) | | 7.50 (5.99-9.33) | | 7.54 (6.21-8.88) | | 7.67 (6.16-9.80) | | 0.450 |
| Platelet count, x 10^9^/L | 235 (197-278) | | 210 (169-248) | | 224 (175-270) | | 224 (174-290) | | **0.031** |
| HbA1c, % | 5.9 (5.4-6.5) | | 6.2 (5.8-6.8) | | 5.8 (5.4-6.9) | | 5.8 (5.3-6.4) | | 0.079 |
| LDL- cholesterol, mg/dL | 114 (81-137) | | 92 (69-123) | | 103 (71-121) | | 88 (64-114) | | **0.021** |
| HDL- cholesterol, mgl/dL | 46 (37-54) | | 45 (37-53) | | 42 (35-51) | | 42 (37-55) | | 0.500 |
| C-reactive protein, mg/L | 6 (3-7) | | 3 (3-8) | | 7 (3-17) | | 11 (3-23) | | **0.001** |
| NT-proBNP, pg/mL | 956 (230-2262) | | 2065 (718-3936) | | 2336 (1433-5165) | | 3933 (1951-7242) | | **0.001** |
| NT-proBNP (eGFR corrected), pg/mL | 748 (337-1643) | | 1102 (429-2356) | | 1276 (579-2506) | | 1975 (1157-4151) | | **0.001** |
| Cardiac troponin I, µg/L | 0.02 (0.02-0.02) | | 0.02 (0.02-0.04) | | 0.02 (0.02-0.04) | | 0.02 (0.02-0.07) | | **0.001** |
| **Medication at discharge**, n (%) |  |  |  |  |  |  |  |  |  |
| ACE-inhibitor | 53 | (43.8) | 72 | (59.0) | 60 | (46.9) | 54 | (44.6) | 0.062 |
| ARB | 40 | (33.1) | 29 | (23.8) | 32 | (25.0) | 41 | (33.9) | 0.172 |
| Beta-blocker | 98 | (81.0) | 104 | (85.2) | 102 | (79.7) | 98 | (81.0) | 0.693 |
| MRA | 24 | (19.8) | 18 | (14.8) | 26 | (20.3) | 19 | (15.7) | 0.565 |
| ARNI | 2 | (1.7) | 1 | (0.8) | 3 | (2.3) | 2 | (1.7) | 0.823 |
| SGLT2-inhibitor | 4 | (3.3) | 5 | (4.1) | 7 | (5.5) | 8 | (6.6) | 0.640 |
| Loop diuretics | 51 | (42.1) | 61 | (50.0) | 77 | (60.2) | 86 | (71.1) | **0.001** |
| Statin | 71 | (58.7) | 90 | (73.8) | 86 | (67.2) | 77 | (63.6) | 0.088 |
| Digitalis | 10 | (8.3) | 5 | (4.1) | 7 | (5.5) | 6 | (5.0) | 0.531 |
| Amiodarone | 6 | (5.0) | 6 | (4.9) | 3 | (2.3) | 4 | (3.3) | 0.647 |
| ASA | 35 | (28.9) | 57 | (46.7) | 59 | (46.1) | 58 | (47.9) | **0.007** |
| P2Y12-inhibitor | 20 | (16.5) | 37 | (30.3) | 33 | (25.8) | 30 | (24.8) | 0.088 |
| DOAC | 50 | (41.3) | 49 | (40.2) | 52 | (40.6) | 44 | (36.4) | 0.861 |
| Vitamin k antagonist | 21 | (17.4) | 16 | (13.1) | 4 | (3.1) | 9 | (7.4) | **0.001** |
| Q, Quartile; NYHA, New York Heart Association; LVEF, left ventricular ejection fraction; IQR, interquartile range; IVSd, Interventricular septal end diastole; mm, millimeter; LVEDD, Left ventricular end-diastolic diameter; TAPSE, tricuspid annular plane systolic excursion; LA, left atrial; VCI, vena cava inferior; CABG, coronary artery bypass grafting; PCI, percutaneous coronary intervention; eGFR, estimated glomerular filtration rate; WBC, white blood cells; HbA1c, glycated hemoglobin; LDL, low-density lipoprotein; HDL, high-density lipoprotein; NT-proBNP, aminoterminal pro-B-type natriuretic peptide; ACE, angiotensin converting enzyme; ARB, Angiotensin II Receptor Blockers; MRA, mineralocorticoid receptor antagonist; ARNI, Angiotensin-receptor-neprilysin-inhibitor; SGLT2, Sodium glucose linked transporter 2; ASA, acetylsalicylic acid; DOAC, directly acting oral anticoagulant.  Level of significance p≤0.05. Bold type indicates statistical significance. | | | | | | | | | |
