# Supplementary material for: D-dimer levels and outcomes in heart failure with mildly reduced ejection fraction
Source: Int J Cardiol Heart Vasc. 2026 Mar 29;64:101915. doi: 10.1016/j.ijcha.2026.101915 (PMC13062528; doi:10.1016/j.ijcha.2026.101915)
Supplement: Supplementary Data 3 [file mmc3.docx]

| **Supplemental Table 3. Follow-up data, primary and secondary endpoints of patients included in analysis 2.** | | | | | | | | | |
| --- | --- | --- | --- | --- | --- | --- | --- | --- | --- |
|  | **Q1**  (*n*=121) | | **Q2**  (*n*=122) | | **Q3**  (*n*=129) | | **Q4**  (*n*=123) | | **p value** |
| **Primary endpoint**, n (%) |  |  |  |  |  |  |  |  |  |
| All-cause mortality, at 30 months | 11 | (9.1) | 24 | (19.7) | 37 | (28.7) | 46 | (37.4) | **0.001** |
| **Secondary endpoints**, n (%) |  |  |  |  |  |  |  |  |  |
| All-cause mortality, in-hospital | 0 | (0.0) | 0 | (0.0) | 1 | (0.8) | 2 | (1.6) | 0.300 |
| Cardiac mortality, in-hospital | 0 | (0.0) | 0 | (0.0) | 1 | (0.8) | 1 | (0.8) | 0.585 |
| Non-cardiac mortality, in-hospital | 0 | (0.0) | 0 | (0.0) | 0 | (0.0) | 1 | (0.8) | 0.387 |
| All-cause mortality, at 12 months | 5 | (4.1) | 11 | (9.0) | 18 | (14.0) | 31 | (25.2) | **0.001** |
| Heart failure-related rehospitalization, at 30 months | 9 | (7.4) | 17 | (13.9) | 35 | (27.3) | 27 | (22.3) | **0.001** |
| Heart failure-related rehospitalization, at 12 months | 6 | (5.0) | 14 | (11.5) | 24 | (18.8) | 21 | (17.4) | **0.005** |
| Cardiac rehospitalization, at 30 months | 28 | (23.1) | 34 | (27.9) | 46 | (35.9) | 41 | (33.9) | 0.114 |
| Coronary revascularization, at 30 months | 7 | (5.8) | 8 | (6.6) | 11 | (8.6) | 10 | (8.3) | 0.804 |
| Acute myocardial infarction, at 30 months | 2 | (1.7) | 3 | (2.5) | 6 | (4.7) | 8 | (6.6) | 0.177 |
| Stroke, at 30 months | 0 | (0.0) | 6 | (4.9) | 3 | (2.3) | 6 | (5.0) | 0.073 |
| MACCE, at 30 months | 19 | (15.7) | 33 | (27.0) | 45 | (34.9) | 57 | (46.3) | **0.001** |
| **Follow-up data**, median (IQR) |  |  |  |  |  |  |  |  |  |
| Hospitalization time, days | 6 (4-8) | | 7 (4-9) | | 7 (4-10) | | 9 (6-15) | | **0.001** |
| ICU time, days | 0 (0-0) | | 0 (0-0) | | 0 (0-0) | | 0 (0-0) | | **0.025** |
| Follow-up time, days | 1548 (981-2110) | | 1050 (605-1705) | | 878 (478-1764) | | 704 (297-1455) | | **0.001** |
| Q, Quartile; MACCE, major adverse cardiac and cerebrovascular events; IQR, Interquartile ratio; ICU, intensive care unit.  Level of significance p≤0.05. Bold type indicates statistical significance. | | | | | | | | | |
