# Supplementary material for: D-dimer levels and outcomes in heart failure with mildly reduced ejection fraction
Source: Int J Cardiol Heart Vasc. 2026 Mar 29;64:101915. doi: 10.1016/j.ijcha.2026.101915 (PMC13062528; doi:10.1016/j.ijcha.2026.101915)
Supplement: Supplementary Data 4 [file mmc4.docx]

| Supplemental Table 4. Multivariable Cox regression analyses with regard to all-cause mortality and heart failure related rehospitalization at 30 months of patients included in analysis 2. | | | | | | |
| --- | --- | --- | --- | --- | --- | --- |
|  | All-cause mortality at 30 months | | | Heart failure related rehospitalization | | |
|  | HR | 95% CI | p value | HR | 95% CI | p value |
| Age | 1.025 | 1.004-1.047 | 0.019 | 0.995 | 0.973-1.018 | 0.688 |
| Sex | 1.559 | 0.975-2.491 | 0.052 | 0.695 | 0.413-1.170 | 0.171 |
| Prior chronic HF | 1.191 | 0.685-1.644 | 0.403 | 1.854 | 1.092-3.148 | 0.022 |
| BMI (per kg/m² increase) | 0.964 | 0.906-0.998 | 0.042 | 1.005 | 0.955-1.059 | 0.837 |
| Diabetes mellitus | 1.061 | 0.677-1.661 | 0.897 | 1.073 | 0.618-1.863 | 0.802 |
| Acute decompensated HF | 1.585 | 0.951-2.643 | 0.077 | 0.988 | 0.539-1.810 | 0.968 |
| Atrial fibrillation | 1.341 | 0.647-2.780 | 0.430 | 1.426 | 0.553-3.681 | 0.463 |
| Anticoagulation | 1.077 | 0.519-2.234 | 0.843 | 1.825 | 0.704-4.730 | 0.216 |
| Ischemic cardiomyopathy | 0.956 | 0.600-1.523 | 0.850 | 1.672 | 0.952-2.937 | 0.074 |
| NYHA functional class | 1.212 | 0.946-1.553 | 0.128 | 2.348 | 1.686-3.270 | 0.001 |
| eGFR (per ml/min/1.73m² increase) | 0.997 | 0.989-1.005 | 0.451 | 0.994 | 0.983-1.006 | 0.321 |
| Hemoglobin (per g/dl increase) | 0.891 | 0.799-0.994 | 0.039 | 1.007 | 0.882-1.150 | 0.914 |
| C-reactive protein | 1.002 | 0.995-1.008 | 0.563 | 1.000 | 0.991-1.009 | 0.990 |
| D-dimer 2^nd^ quartile | 1.704 | 0.719-4.038 | 0.226 | 1.522 | 0.611-3.793 | 0.368 |
| D-dimer 3^rd^ quartile | 2.442 | 1.071-5.564 | 0.034 | 5.421 | 2.313-12.707 | 0.001 |
| D-dimer 4^th^ quartile | 2.880 | 1.280-6.482 | 0.011 | 2.687 | 1.111-6.500 | 0.028 |
| D-dimer 1^st^ quartile | reference group | | | reference group | | |
| HR, hazard ratio; CI, confidence interval; HF, heart failure; BMI, body mass index; NYHA, New York Heart Association; eGFR, estimated glomerular rate..  Level of significance p≤0.05. Bold type indicates statistical significance. | | | | | | |
