# Supplementary material for: D-dimer levels and outcomes in heart failure with mildly reduced ejection fraction
Source: Int J Cardiol Heart Vasc. 2026 Mar 29;64:101915. doi: 10.1016/j.ijcha.2026.101915 (PMC13062528; doi:10.1016/j.ijcha.2026.101915)
Supplement: Supplementary Data 5 [file mmc5.docx]

| **Supplemental Table 5. Baseline characteristics of patients with and without D-dimer testing.** | | | | | |  |
| --- | --- | --- | --- | --- | --- | --- |
|  | **Patient without D-Dimer testing**  (*n*=1058) | | **Patient with D-Dimer testing**  (*n*=1126) | | **p value** |  |
| **Age**, median (IQR) | 65 (55-77) | | 76 (65-82) | | **0.005** | |
| **Male sex**, n (%) | 637 | (63.6) | 737 | (65.5) | 0.368 | |
| **Body mass index,** kg/m^2^, median (IQR) | 26.1 (23.7-29.8) | | 27.1 (24.0-31.1) | | **0.003** | |
| **SBP**, mmHg, median (IQR) | 140 (123-162) | | 143 (127-163) | | 0.378 |  |
| **DBP**, mmHg, median (IQR) | 79 (69-90) | | 79 (69-91) | | 0.265 | |
| **Heart rate**, bpm, median (IQR) | 79 (68-92) | | 82 (69-98) | | **0.001** | |
| **Medical history**, n (%) |  |  |  |  |  | |
| Coronary artery disease | 407 | (38.5) | 489 | (43.4) | **0.019** | |
| Prior myocardial infarction | 220 | (20.8) | 301 | (26.7) | **0.001** | |
| Prior PCI | 252 | (23.8) | 360 | (32.0) | **0.001** | |
| Prior CABG | 104 | (9.8) | 110 | (9.8) | 0.962 | |
| Prior valvular surgery | 46 | (4.3) | 50 | (4.4) | 0.916 | |
| Congestive heart failure | 318 | (30.1) | 423 | (37.6) | **0.001** | |
| Decompensated heart failure <12 months | 98 | (9.3) | 140 | (12.4) | **0.017** | |
| Prior ICD | 20 | (1.9) | 22 | (2-0) | 0.914 | |
| Prior sICD | 3 | (0.3) | 61 | (0.5) | 0.509 | |
| Prior CRT-D | 11 | (1.0) | 21 | (1.9) | 0.109 | |
| Prior Pacemaker | 101 | (9.5) | 99 | (8.8) | 0.541 | |
| Chronic kidney disease | 300 | (28.4) | 379 | (33.7) | **0.007** | |
| Peripheral artery disease | 141 | (13.3) | 112 | (9.9) | **0.014** | |
| Stroke | 172 | (16.3) | 159 | (14.1) | 0.164 | |
| Liver cirrhosis | 24 | (2.3) | 23 | (2.0) | 0.716 | |
| Malignancy | 204 | (19.3) | 131 | (11.6) | **0.001** | |
| COPD | 120 | (11.3) | 143 | (12.7) | 0.330 | |
| **Cardiovascular risk factors,** n (%) |  |  |  |  |  | |
| Arterial hypertension | 830 | (78.4) | 872 | (77.4) | 0.570 | |
| Diabetes mellitus | 381 | (36.0) | 418 | (37.1) | 0.590 | |
| Hyperlipidaemia | 284 | (26.8) | 378 | (33.6) | **0.001** | |
| Smoking | 342 | (32.3) | 454 | (40.3) | **0.001** | |
| Current | 182 | (17.2) | 224 | (19.9) | 0.106 | |
| Former | 160 | (15.1) | 230 | (20.4) | **0.001** | |
| Family history | 69 | (6.5) | 132 | (11.7) | **0.001** | |
| **Comorbidities at index hospitalization**,  n (%) |  |  |  |  |  | |
| Unstable angina | 13 | (1.2) | 86 | (7.6) | **0.001** | |
| STEMI | 94 | (8-9) | 82 | (7.3) | 0.169 | |
| NSTEMI | 72 | (6.8) | 202 | (17.9) | **0.001** | |
| Acute decompensated heart failure | 165 | (15.6) | 319 | (28.3) | **0.001** | |
| Cardiogenic shock | 20 | (1.9) | 33 | (2.9) | 0.114 | |
| Atrial fibrillation | 456 | (43.1) | 460 | (40.9) | 0.287 | |
| Cardiopulmonary resuscitation | 21 | (2.0) | 32 | (2.8) | 0.193 | |
| Stroke | 245 | (23.2) | 53 | (4.7) | **0.001** | |
| bpm, beats per minute; CABG, coronary artery bypass grafting; COPD, chronic obstructive pulmonary disease; CRT-D, cardiac resynchronization therapy with defibrillator; DBP, diastolic blood pressure; IQR, interquartile range; mmHg, millimetres of mercury; (N)STEMI, non-ST-segment elevation myocardial infarction; PCI, percutaneous coronary intervention; Q, Quartile; SBP, systolic blood pressure; (s-)ICD, (subcutaneous) implantable cardioverter defibrillator.  Level of significance p≤0.05. Bold type indicates statistical significance. | | | | | | |
