# Supplementary material for: D-dimer levels and outcomes in heart failure with mildly reduced ejection fraction
Source: Int J Cardiol Heart Vasc. 2026 Mar 29;64:101915. doi: 10.1016/j.ijcha.2026.101915 (PMC13062528; doi:10.1016/j.ijcha.2026.101915)
Supplement: Supplementary Data 6 [file mmc6.docx]

| **Supplemental Table 6. Heart failure related and procedural data comparing patients with and without D-dimer testing.** | | | | | |
| --- | --- | --- | --- | --- | --- |
|  | **Patient without D-Dimer testing**  (*n*=1058) | | **Patient with D-Dimer testing**  (*n*=1126) | | **p value** |
| **Heart failure etiology**, n (%) |  |  |  |  |  |
| Ischemic cardiomyopathy | 547 | (51.7) | 711 | (63.1) | **0.001** |
| Non-ischemic cardiomyopathy | 75 | (7.1) | 74 | (6.6) | 0.632 |
| Hypertensive cardiomyopathy | 119 | (11.2) | 59 | (5.2) | **0.001** |
| Congenital heart disease | 2 | (0.2) | 2 | (0.2) | 0.950 |
| Valvular heart disease | 40 | (3.8) | 56 | (5.0) | 0.174 |
| Tachycardia-associated | 52 | (4.9) | 76 | (6.7) | 0.068 |
| Tachymyopathy | 10 | (0.9) | 28 | (2.5) | **0.006** |
| Pacemaker-induced cardiomyopathy | 10 | (0.9) | 9 | (0.8) | 0.714 |
| Unknown | 213 | (20.1) | 139 | (12.3) | **0.001** |
| **NYHA functional class,** n (%) |  |  |  |  |  |
| I/II | 855 | (80.8) | 730 | (64.8) | **0.001** |
| III | 147 | (13.9) | 263 | (23.4) |  |
| IV | 56 | (5.3) | 133 | (11.8) |  |
| **Echocardiographic data** |  |  |  |  |  |
| LVEF, %, median (IQR) | 45 (45-47) | | 45 (45-47) | | 0.957 |
| IVSd, mm, median (IQR) | 12 (10-13) | | 12 (11-13) | | 0.214 |
| LVEDD, mm, median (IQR) | 49 (44-54) | | 49 (45-54) | | 0.092 |
| TAPSE, mm, median (IQR) | 20 (17-23) | | 20 (17-23) | | 0.504 |
| LA diameter, mm, median (IQR) | 42 (37-48) | | 41 (37-46) | | 0.266 |
| LA area, cm^2^, median (IQR) | 21 (18-26) | | 22 (17-26) | | 0.898 |
| E/A, median (IQR) | 0.8 (0.6-1.2) | | 0.8 (0.6-1.2) | | 0.291 |
| E/E`, median (IQR) | 9.5 (6.6-14.0) | | 9.0 (6.0-13.5) | | 0.330 |
| VCI | 20 (16-25) | | 19 (15-25) | | 0.612 |
| Diastolic dysfunction, n (%) | 745 | (70.4) | 829 | (73.6) | 0.095 |
| Moderate-severe aortic stenosis, n (%) | 110 | (10.4) | 104 | (9.2) | 0.362 |
| Moderate-severe aortic regurgitation, n (%) | 38 | (3.6) | 46 | (4.1) | 0.549 |
| Moderate-severe mitral regurgitation, n (%) | 128 | (12.1) | 134 | (11.9) | 0.887 |
| Moderate-severe tricuspid regurgitation, n (%) | 170 | (16.1) | 174 | (15.5) | 0.693 |
| **Coronary angiography,** n (%) | 319 | (30.2) | 581 | (51.6) | **0.001** |
| No evidence of coronary artery disease | 60 | (18.8) | 115 | (19.8) | 0.401 |
| 1-vessel disease | 55 | (17.2) | 111 | (19.1) |  |
| 2-vessel disease | 78 | (24.5) | 114 | (19.6) |  |
| 3-vessel disease | 126 | (39.5) | 241 | (41.5) |  |
| CABG | 104 | (9.8) | 110 | (9.8) | 0.962 |
| Chronic total occlusion | 42 | (13.2) | 71 | (12.2) | 0.682 |
| PCI, n (%) | 167 | (52.4) | 314 | (54.0) | 0.626 |
| Sent to CABG, n (%) | 22 | (6.9) | 29 | (5.0) | 0.237 |
| **Baseline laboratory values**, median (IQR) |  |  |  |  |  |
| Potassium, mmol/L | 3.9 (3.6-4.2) | | 3.9 (3.6-4-2) | | 0.706 |
| Sodium, mmol/L | 139 (137-141) | | 139 (137-141) | | 0.546 |
| Creatinine, mg/dL | 1.0 (0.8-1.4) | | 1.1 (0.9-1.5) | | **0.004** |
| eGFR, mL/min/1.73 m^2^ | 67 (44-86) | | 64 (44-85) | | **0.049** |
| Hemoglobin, g/dL | 12.2 (10.2-13.9) | | 12.5 (10.6-14.0) | | **0.005** |
| WBC count, x 10^9^/L | 8.17 (6.47-10.2) | | 8.25 (6.43-10.01) | | 0.693 |
| Platelet count, x 10^9^/L | 224 (197-285) | | 229 (177-285) | | 0.385 |
| HbA1c, % | 5.8 (5.5-6.8) | | 5.9 (5.5-6.8) | | 0.188 |
| LDL- cholesterol, mg/dL | 101 (76-126) | | 96 (73-127) | | 0.213 |
| HDL- cholesterol, mgl/dL | 42 (34-53) | | 42 (34-51) | | 0.502 |
| C-reactive protein, mg/L | 15 (4-45) | | 12 (3-42) | | **0.033** |
| NT-proBNP, pg/mL | 2384 (681-6103) | | 2682 (1142-7142) | | 0.076 |
| NT-proBNP (eGFR corrected), pg/mL | 1530 (483-3314) | | 1690 (721-3559) | | 0.104 |
| Cardiac troponin I, µg/L | 0.02 (0.02-0.18) | | 0.03 (0.02-0.18) | | 0.232 |
| **Medication at discharge**, n (%) |  |  |  |  |  |
| ACE-inhibitor | 473 | (46.5) | 585 | (53.6) | **0.001** |
| ARB | 233 | (22.9) | 266 | (24.4) | 0.434 |
| Beta-blocker | 738 | (72.6) | 897 | (82.1) | **0.001** |
| MRA | 123 | (12.1) | 173 | (15.8) | **0.013** |
| ARNI | 10 | (1.0) | 15 | (1.4) | 0.408 |
| SGLT2-inhibitor | 35 | (3.4) | 49 | (4.5) | 0.220 |
| Loop diuretics | 451 | (44.3) | 567 | (51.9) | **0.001** |
| Statin | 679 | (66.8) | 763 | (69.9) | 0.125 |
| Digitalis | 53 | (5.2) | 50 | (4.6) | 0.501 |
| Amiodarone | 24 | (2.4) | 34 | (3.1) | 0.290 |
| ASA | 500 | (49.2) | 563 | (51.6) | 0272 |
| P2Y12-inhibitor | 266 | (26.2) | 402 | (36.8) | **0.001** |
| DOAC | 300 | (29.5) | 390 | (35.7) | **0.002** |
| Vitamin k antagonist | 76 | (7.5) | 74 | (6.8) | 0.534 |
| Q, Quartile; NYHA, New York Heart Association; LVEF, left ventricular ejection fraction; IQR, interquartile range; IVSd, Interventricular septal end diastole; mm, millimeter; LVEDD, Left ventricular end-diastolic diameter; TAPSE, tricuspid annular plane systolic excursion; LA, left atrial; VCI, vena cava inferior; CABG, coronary artery bypass grafting; PCI, percutaneous coronary intervention; eGFR, estimated glomerular filtration rate; WBC, white blood cells; HbA1c, glycated hemoglobin; LDL, low-density lipoprotein; HDL, high-density lipoprotein; NT-proBNP, aminoterminal pro-B-type natriuretic peptide; ACE, angiotensin converting enzyme; ARB, Angiotensin II Receptor Blockers; MRA, mineralocorticoid receptor antagonist; ARNI, Angiotensin-receptor-neprilysin-inhibitor; SGLT2, Sodium glucose linked transporter 2; ASA, acetylsalicylic acid; DOAC, directly acting oral anticoagulant.  Level of significance p≤0.05. Bold type indicates statistical significance. | | | | | |
